# Epidemiology of healthcare-associated ventriculitis and meningitis (HCAVM) and community-acquired meningitis/encephalitis and evaluation of an off-label PCR panel for HCAVM diagnosis

**DOI:** 10.1128/spectrum.02770-25

**Published:** 2025-12-26

**Authors:** Jamie A. Nassur, Katarina M. Schnell, Christine D. Dolon, Laura Walters, Devin Weber, Matthew A. Pettengill

**Affiliations:** 1 Thomas Jefferson University, Sidney Kimmel Medical College 6559, Philadelphia, Pennsylvania, USA; 2 Department of Pathology and Genomic Medicine, Thomas Jefferson University Hospital 23217, Philadelphia, Pennsylvania, USA; 3 Department of Infection Control, Temple University Health – Jeanes Campus, Philadelphia, Pennsylvania, USA; 4 Division of Infectious Diseases, Thomas Jefferson University Hospital 23217, Philadelphia, Pennsylvania, USA; Brigham and Women's Hospital, Boston, Massachusetts, USA

**Keywords:** meningitis, ventriculitis, nosocomial, PCR, rapid

## Abstract

Healthcare-associated ventriculitis and meningitis (HCAVM) is associated with high morbidity and mortality, but rapid diagnostic tests intended to cover microorganisms that typically cause HCAVM are not commercially available. We sought to evaluate the etiology of HCAVM compared to community-acquired meningitis/encephalitis (CAME) at our healthcare system, and to study the utility of repurposing a multiplex molecular panel intended for positive blood cultures for HCAVM cases. Our epidemiologic review demonstrated that HCAVM cases were primarily caused by bacteria and fungi also associated with bloodstream infections, in contrast with CAME cases, which were primarily caused by herpesviruses and *Cryptococcus*. Eighty-two cerebrospinal fluid specimens, including 43 from HCAVM cases, were tested using the BioFire Blood Culture Identification 2 panel, with approximately 90% sensitivity when organisms were seen in the Gram stain but only approximately 50% sensitivity when the stain was negative, compared to standard-of-care testing.

**IMPORTANCE:**

Rapid diagnostic tests are not commercially available that are intended for use in patients who develop infections of their central nervous system (CNS) after CNS surgery or the placement of hardware for CNS issues. Because these types of infections are typically very serious, we attempted to repurpose a commercial assay intended for a different purpose (bloodstream infections) to help generate rapid results for these important CNS infections.

## INTRODUCTION

Healthcare-associated ventriculitis and meningitis (HCAVM) may occur following trauma or neurosurgical intervention involving the central nervous system (CNS) (1, 2). Many HCAVM infections are device-associated, in which a temporary or permanent surgically implanted device—that is, ventriculostomy, lumbar drain, or ventricular shunt, among others—becomes infected through microbial contamination or colonization (3). Historically, Gram-positive skin flora, including *Staphylococcus* species and *Cutibacterium acnes,* have been the predominant causative agents of HCAVM (4–8). More recently, a shift toward Gram-negative bacilli and multidrug-resistant organisms as emerging pathogens in HCAVM has been reported in the literature (4, 5, 7, 9, 10). HCAVM is associated with significant adverse outcomes and in-hospital mortality, making rapid and accurate pathogen identification critical for timely, targeted antimicrobial treatment (1, 7).

The gold standard for microbiologic diagnosis of HCAVM is cerebrospinal fluid (CSF) culture and Gram stain; however, the sensitivity can be low, especially with antibiotic administration prior to specimen collection (1, 3). Nucleic acid amplification tests have aided in the rapid identification of microbial pathogens while minimizing false negatives that may result from prior antibiotic treatment (1, 3). Currently, the only Food and Drug Administration (FDA)-approved multiplex polymerase chain reaction (PCR) assays to detect pathogens in CSF are the BioFire FilmArray Meningitis/Encephalitis (M/E) panel and the QIAstat-Dx Meningitis/Encephalitis panel, both of which target community-acquired meningitis/encephalitis (CAME) caused by select bacterial, fungal, and viral pathogens in CSF obtained from lumbar punctures (LP). These panels do not cover the major bacterial pathogens responsible for HCAVM and are not FDA-approved for CSF specimens obtained from indwelling neurosurgical devices.

The BioFire Blood Culture Identification 2 (BCID2) panel is an FDA-approved multiplex PCR designed for the rapid detection of 43 targets, including bloodstream pathogens and AMR genes (11). Many organisms responsible for HCAVM are included in the BCID2 panel, including Gram-positive skin flora, Gram-negative bacilli, and multidrug-resistant pathogens. This raises the possibility of repurposing the BCID2 panel for rapid pathogen detection in CSF samples for patients with concern for healthcare- or device-associated CNS infection. A study assessing the use of the initial FilmArray Blood Culture Identification Panel (BCID) for HCAVM CSF specimens found a sensitivity of 77.4% and specificity of 100%, supporting the potential utility of a similar panel in the diagnosis of HCAVM (12).

The objectives of this study are twofold: (i) to provide epidemiologic data of organisms detected in community-acquired (CAME) versus device-associated (HCAVM) CNS disease from an academic hospital system with a large neurosurgical patient population and (ii) to assess the performance of the BCID2 panel for the detection of organisms in CSF specimens.

## MATERIALS AND METHODS

### Epidemiology of CAME and HCAVM

This study was conducted at a large university hospital system with a specialized neuroscience hospital that oversees a high volume of neurosurgical patients. Epidemiological evaluation of HCAVM and CAME etiologies was performed with an IRB-approved study # iRISID-2023-1943. We reviewed the results of CSF microbiologic diagnostic tests performed in-house in our health system from April 2017 through March 2024. Electronic medical record (EMR) data produced 13,784 cultures and Gram stains of CSF from 8,499 unique patients, 1,109 cryptococcal antigen assays from 990 unique patients, and 5,844 molecular M/E panels from 5,254 unique patients. We included only abnormal values from the in-house tests for further chart review to determine the causative organisms, CNS disease categorization (HCAVM vs CAME), and CSF parameters for all new patient diagnoses. New diagnoses were defined as any abnormal value for a specimen with an organism not identified in the prior 30 days from the same patient. HCAVM cases were defined as patients with CSF collected from an implanted device or from an LP but with documented history of CNS surgery or trauma in the previous 6 months. CAME cases were defined as patients with CSF collected by LP and with no history of recent CNS surgery or trauma. Of note, we could not rely on the specimen source listed in the EMR’s diagnostic test result to determine whether a specimen was collected via LP or not, as the EMR allowed for both specimen type and source to be labeled as “CSF.” Additionally, we knew of cases with the source labeled incorrectly, and we encountered some HCAVM cases with CSF collected via LP.

### BCID2 performance for CSF specimens

Patient CSF samples included in the BCID2 performance study were identified based on culture or PCR standard-of-care (SOC) testing conducted in the Thomas Jefferson University Hospital Clinical Microbiology Laboratory. Positive CSF samples included those with a positive culture or PCR result on the BioFire FilmArray M/E panel. Of note, SOC cultures included the use of 5% sheep’s blood agar, chocolate agar, MacConkey agar, CDC anaerobic agar, and thioglycolate broth (with CDC Ana and thioglycolate held 5 days). Gram stains from CSF were performed using a cytospin concentration slide. Positive samples were then further classified based on whether organisms were identified on specimen smear review of the Gram stain. CSF specimens that were negative with in-house testing or positive for organisms not covered by the BCID2 panel were selected to serve as controls. All positive and control CSF specimens were run on the BCID2 panel per the manufacturer’s instructions, except that CSF was used instead of the approved specimen type of blood.

To evaluate the detection capacity of the BCID2 for CSF specimens, the limit of detection (LOD) for a sample of organisms covered by the BCID2 panel (including Gram-positive and -negative bacteria, fungi, and drug-resistant organisms) was performed. Ten-fold serial dilutions of the organisms (ATCC strains) were prepared and suspended in trypticase soy broth to a concentration of approximately 1.0 McFarland and then serially diluted using a pool of confirmed culture-negative patient CSF specimens. The organisms included were: MRSA ATCC 43300, *Proteus mirabilis* ATCC 12453, *Pseudomonas aeruginosa* ATCC 27853, *Klebsiella pneumoniae* ATCC BAA1705 (positive for KPC), *Streptococcus pneumoniae* ATCC 49619, *Candida albicans* ATCC 14053, and *Cryptococcus neoformans* ATCC 66031. We performed an additional 10-fold serial dilution on the already serially diluted samples and cultured these on sheep’s blood agar, MacConkey agar, or Sabouraud agar. For plates with approximately 1 to 150 colonies, we manually counted the colonies and performed a back-calculation to determine the organism concentration for each dilution. The dilutions were then run on the BCID2 panel to determine the lowest concentration at which the organism could be identified.

Data collected for each CSF specimen included the unique specimen identification number, BCID2 result, culture result, smear review of the Gram stain, M/E panel result, method of collection, and CSF fluid characteristics, including the white blood cell (WBC) count and differential. Analyses were conducted using Excel to determine the sensitivity and specificity of the BCID2 panel in detecting organisms in CSF samples.

## RESULTS

The epidemiologic review yielded 814 diagnostic events from 764 unique patients. Eight events were excluded due to having insufficient EMR data for categorization. Of the remaining 806 events, 542 were from CAME cases and 264 were from HCAVM cases, some of which were polymicrobial (Table 1). CAME cases were expectedly dominated by herpes family viruses, *Streptococcus pneumoniae*, and *Cryptococcus neoformans*. HCAVM cases were more diverse, with representation of many organisms that are common constituents of skin or gastrointestinal flora. The most common causes of HCAVM in our data were *Cutibacterium acnes* (55 cases), *Enterobacterales* (52), *Staphylococcus epidermidis* (47), and *Staphylococcus aureus* (33).

**TABLE 1:** Comparison of frequency of detection of pathogens in clinical cases of CAME and HCAVM^*j*^

CAME	*n*	WBC	HCAVM	*n*	WBC
VZV	66	83	*Cutibacterium acnes*	55	5
HHV-6	49	1	*Enterobacterales* ^*b*^	52	1,941
*Streptococcus pneumoniae*	47	1,154	*S. epidermidis*	47	190
*Cryptococcus neoformans*	39	65	*Staphylococcus aureus*	33	269
HSV-2	34	434	Other Gram-positive^*c*^	17	31
Enterovirus	30	68	*Streptococcus* spp.^*d*^	14	1,995
HSV-1	22	49	*P. aeruginosa*	13	1,286
Other^*a*^	13	27	Other *Staphylococcus*^*e*^	11	87
*Haemophilus influenzae*	12	1,498	Other viruses^*f*^	8	87
CMV	9	7	*Candida* spp.^*g*^	8	48
*Streptococcus agalactiae*	8	1,279	Other Gram-negative^*h*^	8	2,615
Parechovirus	5	18	HHV-6	6	4
*Listeria monocytogenes*	5	440	Other fungi^*i*^	6	6
*Escherichia coli*	5	2,806	*Enterococcus faecalis*	5	231

^*a*^ LP Other included MSSA (4), MRSA (2), *S. epidermidis *(1), *S. mitis/oralis *(1), *S. pyogenes *(1), *N. meningitidis *(1), *P. aeruginosa *(1), *C. freundii *(1), and* P. mirabilis *(1).

^*b*^ *K. pneumoniae *(17), *E. cloacae *(10), *E. coli *(8), *K. aerogenes* (5), *K. oxytoca *(3), *S. marcescens *(3), other *Enterobacterales* (6).

^*c*^ *E. faecium *(3), *L. monocytogenes *(2), *B. cereus *(2), other *Bacillus* spp. (2), *Lactobacillus* spp. (2), *Corynebacterium* spp. (2), *Brevundimonas diminuta *(1), *Rothia dentocariosa *(1), *Eggerthella* spp. (1), *Clostridium tertium *(1).

^*d*^ *S. mitis/oralis *(5), *S. anginosus *group (4), *S. pneumoniae *(3), *S. agalactiae *(1), *S. dysgalactiae *(1).

^*e*^ *S. capitis *(3), other coagulase-negative staphylococci (8).

^*f*^ CMV (4), VZV (3), HSV-2 (1).

^*g*^ *C. albicans *(5), *C. parapsilosis *(2), *C. tropicalis *(1).

^*h*^ *Bacteroides* spp. (2), *Porphyromonas* spp. (1), *Capnocytophaga ochracea *(1), *Acinetobacter radioresistens *(1), *N. meningitidis *(1), *N. flavescens/subflava *group (1), *Moraxella osloensis *(1).

^*i*^ *C. neoformans *(3), *Aspergillus fumigatus *(1), *Coccidioides posadasii *(1), other mold not identified (1).

^*j*^ *n*, total cases, including if detected in polymicrobial infection; WBC, median white blood cell count from cases where the indicated organism was the only organism detected.

### CSF specimen characteristics

Between January 2018 and May 2024, 108 patient CSF specimens, including 82 that were culture- or M/E panel-positive for organisms on the BCID2 panel, and 26 control specimens, were identified, frozen at −80°C, and run on the BCID2 panel. The 26 control specimens included 20 LP collections from patients with CNS disease but for whom an infectious process was considered unlikely, per chart review, and 6 specimens were non-LP CSF collections which were negative by SOC testing (4) or positive for an off-panel target (*C. acnes*, 2). Of the positive specimens, 43 (52%) were Gram-positive organisms, 27 (33%) were Gram-negative organisms, and 12 (15%) were fungal organisms. On smear review of the Gram stain, 18 (42%) of the Gram-positive organisms, 14 (52%) of the Gram-negative organisms, and 9 (75%) of the fungal organisms were seen on Gram stain. Of the 33 organisms that the BCID2 panel can detect, we did not have positive patient specimens available to evaluate 10 of the organisms, including *Listeria monocytogenes, Streptococcus pyogenes, Acinetobacter calcoaceticus-baumannii complex, Bacteroides fragilis, Salmonella species, Neisseria meningitidis, Candida auris, Candida glabrata,* and *Candida krusei*.

The overall sensitivity of the BCID2 panel to detect organisms in patient CSF samples was 67% compared to SOC. When the specimens were categorized based on whether organisms were seen on Gram stain smear review, specimens seen on Gram stain had a sensitivity of 88% compared to 48% for specimens with no organisms seen. The sensitivity of the BCID2 panel was also determined based on whether organisms were Gram-positive, Gram-negative, or fungal. For specimens with organisms seen on Gram stain smear review, those with Gram-negative organisms had the highest sensitivity at 100%, followed by fungal organisms at 89% and Gram-positive organisms at 76%. Sensitivity was similar for CSF specimens associated with CAME or HCAVM cases. Modest improvements in sensitivity were observed when excluding cases in which the attending infectious disease physician considered that the SOC result represented contamination (Table 2).

**TABLE 2:** Technical sensitivity compared to SOC for BCID2 performed on CSF from CAME and HCAVM^*m*^

	CAME			HCAVM			All		
	Gram stain +	Gram stain −	All	Gram stain +	Gram stain −	All	Gram stain +	Gram stain −	All
Gram-positive	4/5^*a*^	9/19^*b*^	13/24	9/12^*g*^	2/7^*h*^	11/19	13/17	11/26	24/43
Gram-negative	1/1^*c*^	4/7^*d*^	5/8	13/13^*i*^	4/6^*j*^	17/19	14/14	8/13	22/27
Fungal	5/6^*e*^	0/1^*f*^	5/7	3/3^*k*^	1/2^*l*^	4/5	8/9	1/3	9/12
All	10/12 (**83%**)	13/27 (**48%**)	23/39 (**59%**)	25/28 (**89%**)	7/15 (**47%**)	32/43 (**74%**)	35/40 (**88%**)	20/42 (**48%**)	55/82 (**67%**)
All, excl. prob. contam	10/11 (**91%**)	13/22 (**59%**)	23/33 (**70%**)	25/28 (**89%**)	7/13 (**54%**)	32/41 (**78%**)	35/39 (**90%**)	20/35 (**57%**)	55/74 (**74%**)

^*a*^ *S. agalactiae* (1/1), *S. dysgalactiae* (1/1), *S. pneumoniae* (1/1), *E. faecium* (1/1), and *S. capitis* (0/1).

^*b*^ *S. pneumoniae* (8/10), *S. epidermidis* (0/3—two SOC-positives were thioglycolate broth only), *S. agalactiae* (0/2, both SOC-positives were M/E panel only, culture negative), MRSA (1/1), MSSA (0/1), *S. lugdunensis* (0/1, SOC-positive was thioglycolate broth only), *S. hominis* (0/1).

^*c*^ *Pseudomonas aeruginosa* (1/1).

^*d*^ *H. influenzae* (4/5), *S. maltophilia* (0/1, SOC-positive was thioglycolate broth only), *P. mirabilis* (0/1).

^*e*^ *Cryptococcus neoformans* (5/6).

^*f*^ *C. neoformans* (0/1).

^*g*^ *S. epidermidis* (5/5), MSSA (2/3), *S. mitis*/*oralis* (1/1), *S. hominis* (1/1), *E. faecalis* (0/1), *E. faecium* (0/1).

^*h*^ *S. epidermidis* (2/3), MRSA (0/1), MSSA (0/1), *E. faecalis* (0/1), *S. mitis*/*oralis* (0/1).

^*i*^ *K. pneumoniae* (5/5, 3 were CTX-M also detected), *K. aerogenes* (2/2), *Proteus* spp. (2/2, both *P. mirabilis*), *Serratia marcescens* (2/2), *K. oxytoca* (1/1), *Enterobacterales* target only (1/1, *Providencia rettgeri*).

^*j*^ *P. aeruginosa* (2/2), *K. pneumoniae* CTX-M (1/1), *K. aerogenes* (1/1), *E. cloacae* (0/1), *S. maltophilia* (0/1, SOC-positive culture was 1 colony only).

^*k*^ *C. albicans* (1/1), *C. tropicalis* (1/1), *C. parapsilosis* (1/1).

^*l*^ *C. albicans* (1/1), *C. tropicalis* (0/1).

^*m*^ Sensitivity of BCID2 on direct CSF for detecting organisms positive by SOC testing separated by CAME/HCAVM status, Gram stain results, and organism class. Sensitivity also calculated excluded cases for which an infectious disease physician specified in a chart note that the SOC organism was considered a contaminant and not treated (bottom row). Organisms in each category were (number detected out number in group shown in parentheses).

Specificity for the BCID2 assay performed on direct CSF samples was 100%, which included 19 (73%) specimens negative by SOC and 7 (27%) specimens positive for *Cutibacterium acnes*, an organism not detected by the BCID2 panel. Additionally, there were no false-positive results for other targets among specimens with positive results, either by the BCID2 panel or SOC.

Eleven CSF specimens run on BCID2 had AMR genes identified, including four CTX-M (extended spectrum β-lactamase), four mec A/C (methicillin resistance gene), two mec A/C *and* MREJ (methicillin resistance mec cassette/orfX right extremity junction—associates mecA with *S. aureus*), and one van A/B (which causes vancomycin resistance in some enterococci). All AMR genes detected correlated with SOC susceptibility testing results. For organisms detected by the BCID2, there were no cases where resistance genes predicted by SOC were missed by the BCID2; however, the overall number of specimens positive for AMR genes was low. Additional characteristics and organisms identified in the CSF specimens can be seen in Table 2.

### BCID2 panel LOD

An exact determination of the LOD would have been cost-prohibitive due to the high cost of BCID2 reagents. Thus, we evaluated several organisms for an approximate LOD using serially diluted organisms in a remnant pooled CSF, which was confirmed to be negative for organisms by running a small portion of the pool in the BCID2 panel. *S. aureus*, *S. pneumoniae*, *K. pneumoniae*, and *P. aeruginosa* were detected between approximately 1.4 × 10^3^ and 2.1 × 10^4^ CFU/mL but not in the samples 10-fold diluted from those concentrations. Fungi were detected at lower levels*—C. albicans* at 2.0 × 10^2^ and *C. neoformans* at 5.4 × 10^2^. Interestingly, *P. mirabilis* required significantly more organisms for detection at 1.6 × 10^5^ CFU/mL. The values found in our limited study correlate well with those shown in the publicly available manufacturer’s instructions for use.

## DISCUSSION

The etiologies of CAME and HCAVM differ substantially, with little overlap in the causative pathogens. While there are rapid diagnostic assays available for CAME, there are not currently HCAVM-targeted assays commercially available. Given the presence of a large neuroscience hospital in addition to several academic and community hospitals in our clinical setting, we see a considerable number of HCAVM cases. Thus, we sought to determine if repurposing a blood culture-based rapid identification assay could have utility in this space. Although it was our impression that a blood assay would adequately detect the causative organisms of HCAVM, there is limited study data available to address this question, and we lacked supporting local data.

In reviewing more than 800 cases of CAME and HCAVM at our institution, several interesting issues were raised beyond the epidemiologic differences alone. Positive cultures may represent contamination either due to inadequate collection and storage methods or handling during plating or culture reading (i.e., in open air and not in a biosafety cabinet). In our review of cases, care providers readily dismissed possible skin flora contaminants in CAME cases. However, dismissal of skin flora contaminants proved difficult for HCAVM cases since these organisms frequently cause HCAVM. Despite this, it is highly likely that some of the HCAVM cases with skin flora are falsely positive. Of note, for our HCAVM cases, the median WBC count in CSF from 52 cases growing only *C. acnes* was 5 WBC, and of 40 cases growing only *S. epidermidis,* 6 cases had no pleocytosis. Among the potential CAME cases we identified, 203 samples were excluded as contaminants due to growth of skin flora only and documentation by a consulted ID physician deeming the result clinically insignificant. For these 203 cases, the median CSF WBC count was 2.

Additionally, although likely not positive due to contamination or a true technical false-positive, human herpesvirus 6 (HHV-6) positive results are rarely clinically relevant. HHV-6 may cause encephalitis, primarily in immunocompromised patients, but it is more frequently detected spuriously due to insertion of the viral genome into human chromosome telomeres, which can be transmitted to progeny as germline-encoded virus and thus detectable in any cells, including WBCs that end up in CSF (13). Among 56 cases where HHV-6 was detected in our study, only 9 were treated (5 with ganciclovir and 4 with foscarnet). For two of the treated cases, the infectious disease attending physician specifically did not recommend treatment. Of all the BioFire M/E panels run during our study period, 0.96% (56/5,844) were positive, which is consistent with the estimated 1% chromosomal integration rate in humans (14).

Our results also revealed we had a relatively high number of varicella zoster virus (VZV) detections among our CAME cases. This is likely due in part to the fact that our academic medical center microbiology lab performs all of the microbiology testing for a large specialty ophthalmology hospital. Of the 69 cases of VZV (CAME or HCAVM) in our study, 25 cases included ophthalmologic symptoms in their initial presentation.

As noted previously, the BCID2 panel is FDA-approved for performance on positive blood culture bottle fluid where a relatively high concentration of organism would be expected. Our limited evaluation of the LOD of organisms suspended in CSF suggests the BCID2 assay is not designed to detect very low quantities of organism, possibly to reduce the generation of false-positive results when low levels of target nucleic acids are present. Of note, we did observe a higher approximate LOD for *Proteus mirabilis*—an organism for which there had been a previous issue with BD BACTEC blood culture bottles containing residual non-viable nucleic acid that led to false-positive detection with the BCID, resulting in the manufacturer potentially adjusting the LOD for this target in BCID2. Given this context, it is not surprising that we observed higher sensitivity when the organisms detected in SOC testing were observed in the Gram stain compared to stain negative specimens, which have a lower burden of organism (Table 2). The sensitivity of the BCID2 to detect organisms present was approximately 90% in cases where the organism was seen on the Gram stain, and only about 50% when the Gram stain was negative. Performance was moderately better for Gram-negative bacteria, for which 100% were BCID2 positive (14/14) when observed in the Gram stain, than for other organism groups. Performance was also modestly better when excluding cases for which the SOC result was considered a contaminant by the attending infectious diseases physician. Of the organisms detected by SOC testing in our CAME epidemiological data set, 96% are included in the M/E multiplex panel, while 38% are included in the BCID2 panel. Of the organisms detected by SOC testing in our HCAVM epidemiological data set, only 8% are included in the M/E multiplex panel, while 67% are included in the BCID2 panel. The missing coverage is primarily due to *C. acnes* not being included in the BCID2 panel. Based on the performance data shown herein, we proceeded with the use of the BCID2 assay on CSF at our institution in the following two circumstances: organisms were seen in the Gram stain (and if LP collection and routine meningitis molecular ordered it did not identify the organisms seen), or such testing was requested by an ID physician and the specimen had a WBC count of ≥100 cells.

Of note, we were surprised by the low sensitivity of Gram stain for detecting organisms causing CAME and HCAVM. In our data set, we recorded whether the organisms detected by SOC testing were observed in the Gram stain. For CAME cases with bacterial or fungal organisms detected, only 33 of 128 (26%) were observed in the Gram stain. For HCAVM cases, the detection rate was only modestly higher at 91 of 265 (34%). These results, in conjunction with findings on the performance of the BCID2 for specimens with and without organisms seen on Gram stain, suggest that while the BCID2 assay for HCAVM cases may be helpful in quickly identifying organisms observed on Gram stain, negative results are not clinically actionable.

Our study has several limitations, some of which were already noted. The epidemiological review could not feasibly produce a denominator of total samples from CAME and HCAVM cases as the number of patient charts to review to determine true source and clinical context would have been too high. We only reviewed cases with an abnormal (i.e., positive) value and thus cannot comment on the rate at which various positive results occurred or differed between CAME and HCAVM. Additionally, we only reviewed results from in-house testing, which does not include less common causes of CAME such as arboviruses or parasites. We also ran relatively few specimens that were negative by SOC testing and thus could not determine how many of these cases, either due to prior antibiotic use or other reasons, may have been detectable with BCID2. There are other multiplex molecular assays that are FDA-approved for blood cultures, and these may perform differently with direct specimens than what we observed with the BCID2 panel. One such assay also includes coverage of *C. acnes* (as part of a contamination rule-out procedure) (15).

Overall, our data suggest that repurposing a multiplex panel intended for blood cultures offers reasonable coverage for the organisms observed HCAVM cases and offers high specificity but only moderate sensitivity when organisms are at a high enough concentration to be observed in the Gram stain and low sensitivity for Gram stain-negative specimens. HCAVM patients would benefit from testing specifically designed to provide rapid and more sensitive results for this clinical indication.
